# Supplementary material for: Glutathione synthesis in the mouse liver supports lipid abundance through NRF2 repression
Source: Nat Commun. 2024 Jul 21;15:6152. doi: 10.1038/s41467-024-50454-2 (PMC11271484; doi:10.1038/s41467-024-50454-2)
Supplement: Supplementary file 1 — Supplementary Information [file 41467_2024_50454_MOESM1_ESM.pdf]

Figure S1_related to Figure 1

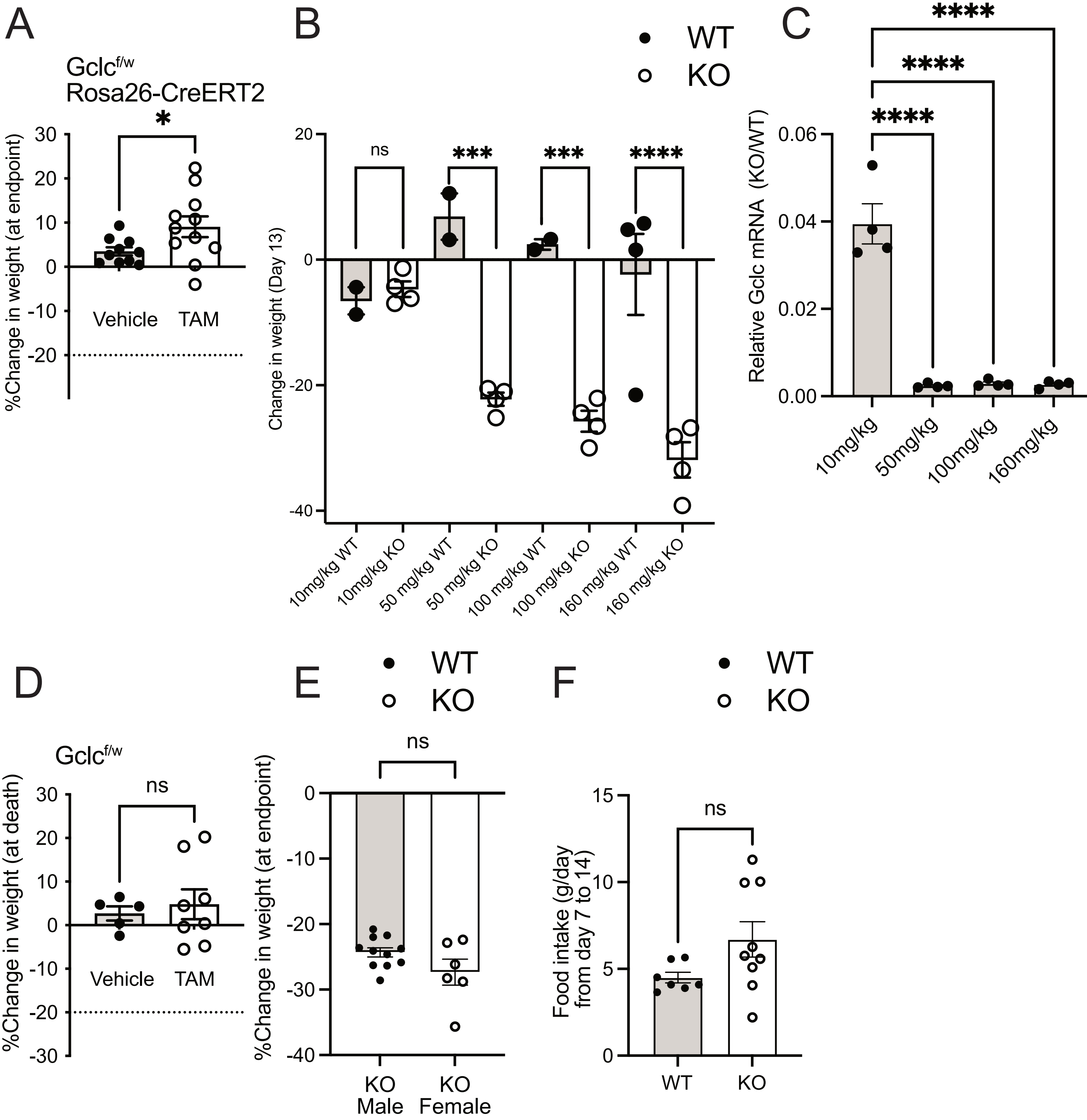

Figure S1. GSH depletion causes weight loss and reduced survival in mice. Related to Figure 1.

(A) Percent change in body weight in *Gclc*$^{f/w}$ Rosa26-CreERT2 mice treated with either vehicle (corn oil) (*n*=10) or tamoxifen (*n*=11). The dotted line indicates a 20% cutoff weight change for humane endpoints. An unpaired two-tailed t-test was used to determine statistical significance (Vehicle vs. TAM P value = 0.0466).

(B) Percent change in body weight at endpoint between WT (*n*=2-4) and KO (*n*=4) mice treated with varying tamoxifen doses (10, 50, 100, and 160) mg/kg body weight. A two-way ANOVA with subsequent Šidák's multiple comparisons test was used to determine statistical significance (WT vs. KO: 10mg/kg P value = 0.9950, 50mg/kg P value = 0.0002, 100mg/kg P value = 0.0002, 160mg/kg P value < 0.0001)

(C) Relative expression of *Gclc* mRNA in the liver from the KO (*n*=4) compared to WT (*n*=4) mice treated with different doses of tamoxifen (10, 50, 100, and 160 mg/kg body weight). A one-way ANOVA with subsequent Dunnett's multiple comparisons test was used to determine statistical significance (10mg/kg vs. 50mg/kg P value < 0.0001, 10mg/kg vs. 100mg/kg P value < 0.0001, 10mg/kg vs. 160mg/kg P value < 0.0001)

(D) Percent change in body weight at the endpoint in *Gclc*$^{f/w}$ mice treated with vehicle (corn oil) (*n*=4) or tamoxifen (*n*=8). An unpaired two-tailed t-test was used to determine statistical significance (Vehicle vs. TAM P value = 0.6559).

(E) Percent change in body weight at the endpoint in male (*n*= 11) and female (*n*=5) KO mice. An unpaired two-tailed t-test was used to determine statistical significance (KO male vs. KO Female P value = 0.1002).

(F) Consumption of food by WT (*n*=7) and KO (*n*=5) mice over time. An unpaired two-tailed t-test was used to determine statistical significance (WT vs. KO P value = 0.0875).

Indicated *n* values represent biologically independent samples from mice. Data are shown as mean ±SEM. ns = not significant, * P value < 0.05, ** P value < 0.01, *** P value < 0.001, **** P value < 0.0001.

Figure S2_related to Figure 2

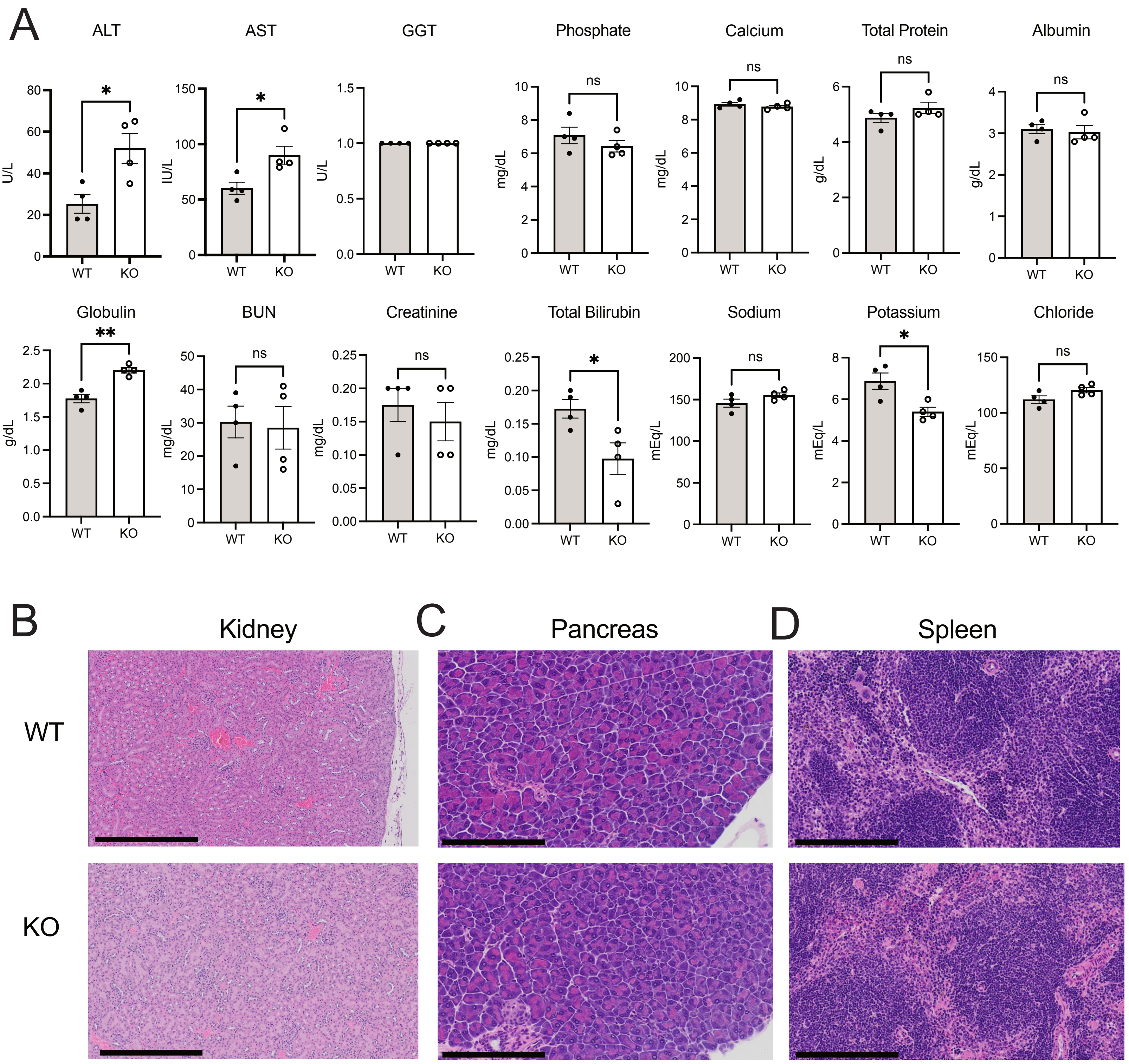

Figure S2. Ablation of GSH synthesis in animals does not cause damage to multiple tissues. Related to Figure 2.

(A) Biochemical analysis of annotated serum biomarkers from WT (*n*=4) and KO (*n*=4) mice. An unpaired two-tailed t-test was used to determine statistical significance (WT vs. KO: ALT P value = 0.0197, AST P value = 0.0222, Phosphate P value = 0.3221, Calcium P value = 0.3250, Total Protein P value = 0.2124, Albumin P value = 0.7111, Globulin P value = 0.0013, BUN P value = 0.8341, Creatinine P value = 0.5370, Total Bilirubin P value = 0.0348, Sodium P value = 0.1430, Potassium P value = 0.0163, Chloride P value = 0.0855).

(B-D) Representative H&E-stained slides of the (B) kidney, (C) pancreas, and (D) spleen from WT mice (top) and KO (bottom) mice. Scale bars = (B) 500 µm, (C-D) 200 µm. Data shown is representative of at least 3 replicates.

Indicated *n* values represent biologically independent samples from mice. Data are shown as mean ±SEM. ns = not significant, * P value < 0.05, ** P value < 0.01, *** P value < 0.001, **** P value < 0.0001.

Figure S3_related to Figure 2

A

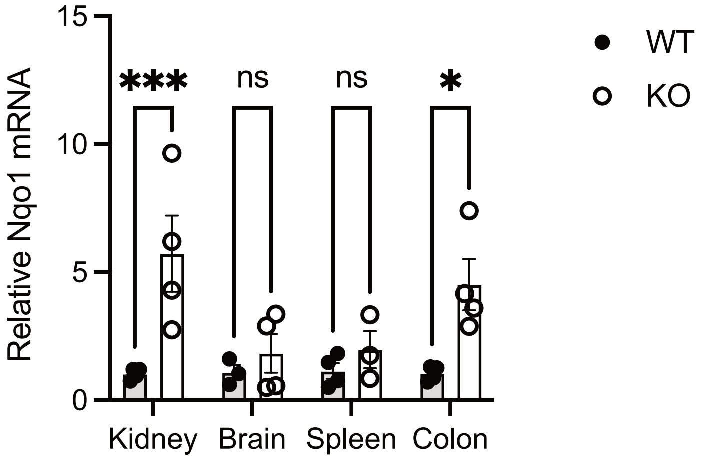

B

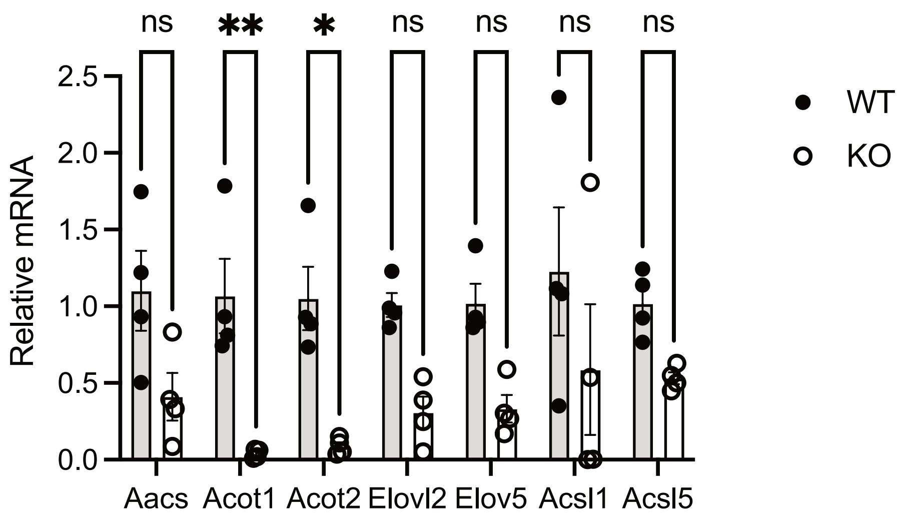

C

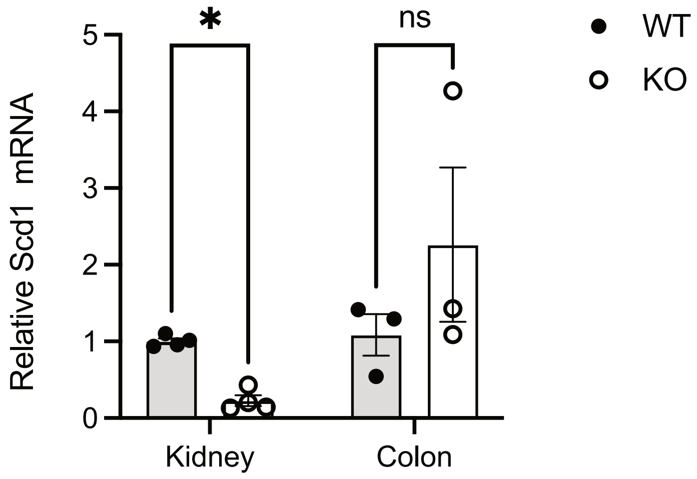

Figure S3. Ablation of GSH synthesis induces expression of NRF2-target genes across several tissues but only decreases the expression of lipogenic genes in select tissues. Related to Figure 2.

(A) Relative expression of *Nqo1* mRNA in the kidney, brain, spleen, and colon from WT (*n*=4) and KO (*n*=4) mice. Expression levels were normalized to the expression of the reference gene *Rps9*. A two-way ANOVA with subsequent Šidák's multiple comparisons test was used to determine statistical significance (WT vs. KO: Kidney P value = 0.0009, Brain P value = 0.9493, Spleen P value = 0.9269, Colon P value = 0.0149)

(B) Relative mRNA expression of annotated lipogenic genes in the liver of WT (*n*=4) and KO (*n*=4) mice. Expression levels were normalized to the expression of the reference gene *Rps9*. A two-way ANOVA with subsequent Šidák's multiple comparisons test was used to determine statistical significance (WT vs. KO: *Aacs* P value = 0.1536, *Acot1* P value = 0.0078, *Acot2* P value = 0.0143, *Elovl2* P value = 0.1411, *Elovl5* P value = 0.1555, *Acsl1* P value = 0.2193, *Acsl5* P value = 0.5422)

(C) Relative expression of *Scd1* mRNA in the kidney and colon from Gclc WT (*n*=4) mice and Gclc KO (*n*=4) mice. Expression levels were normalized to the expression of the reference gene *Rps9*. Multiple unpaired t-tests were used to determine statistical significance (WT vs. KO: Kidney P value = 0.000064, Colon P value = 0.162903).

Indicated *n* values represent biologically independent samples from mice. Data are shown as mean ±SEM. ns = not significant, * P value < 0.05, ** P value < 0.01, *** P value < 0.001, **** P value < 0.0001.

Figure S4_related to Figure 3

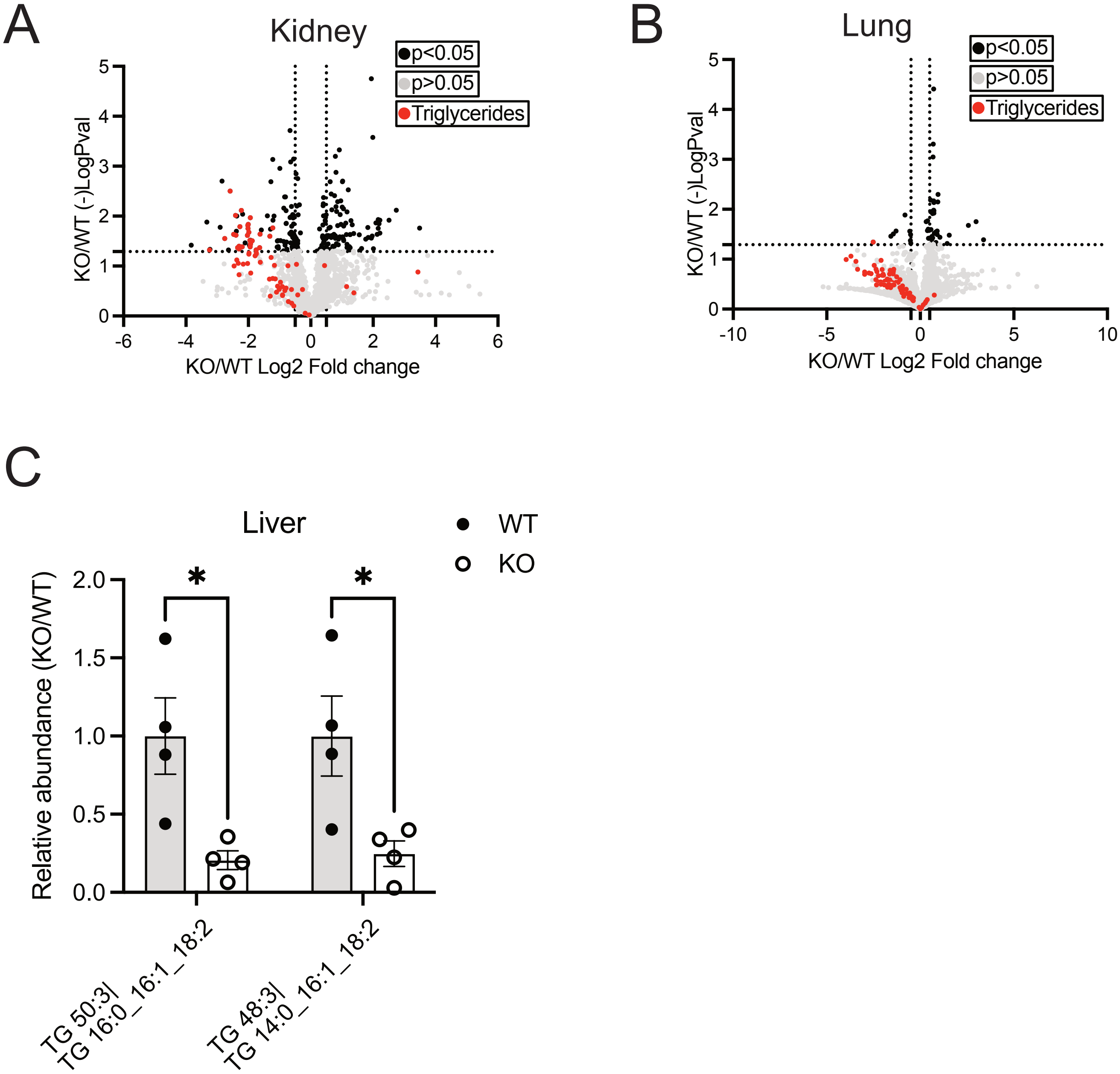

Figure S4. Blocking GSH synthesis lowers the abundance of triglycerides in tissues. Related to Figure 3.

(A-B) Fold change of lipid species from the kidney (A) and lung (B) of Gclc WT and Gclc KO mice. Black data points = lipid species with a p <0.05 and log2 fold change > 1. Red data points = triglycerides.

(C) Relative abundance of annotated triglyceride species in the liver of WT (*n*=4) and KO (*n*=4) mice. A two-way ANOVA with subsequent Šidák's multiple comparisons test was used to determine statistical significance (WT vs. KO: TG 50:3|TG 16:0_16:1_18:2 P value = 0.0203, TG 48:3|TG 14:0_16:1_18:2 P value = 0.0272)

Indicated *n* values represent biologically independent samples from mice. Data are shown as mean ±SEM. ns = not significant, * P value < 0.05, ** P value < 0.01, *** P value < 0.001, **** P value < 0.0001.

Figure S5_related to Figure 4

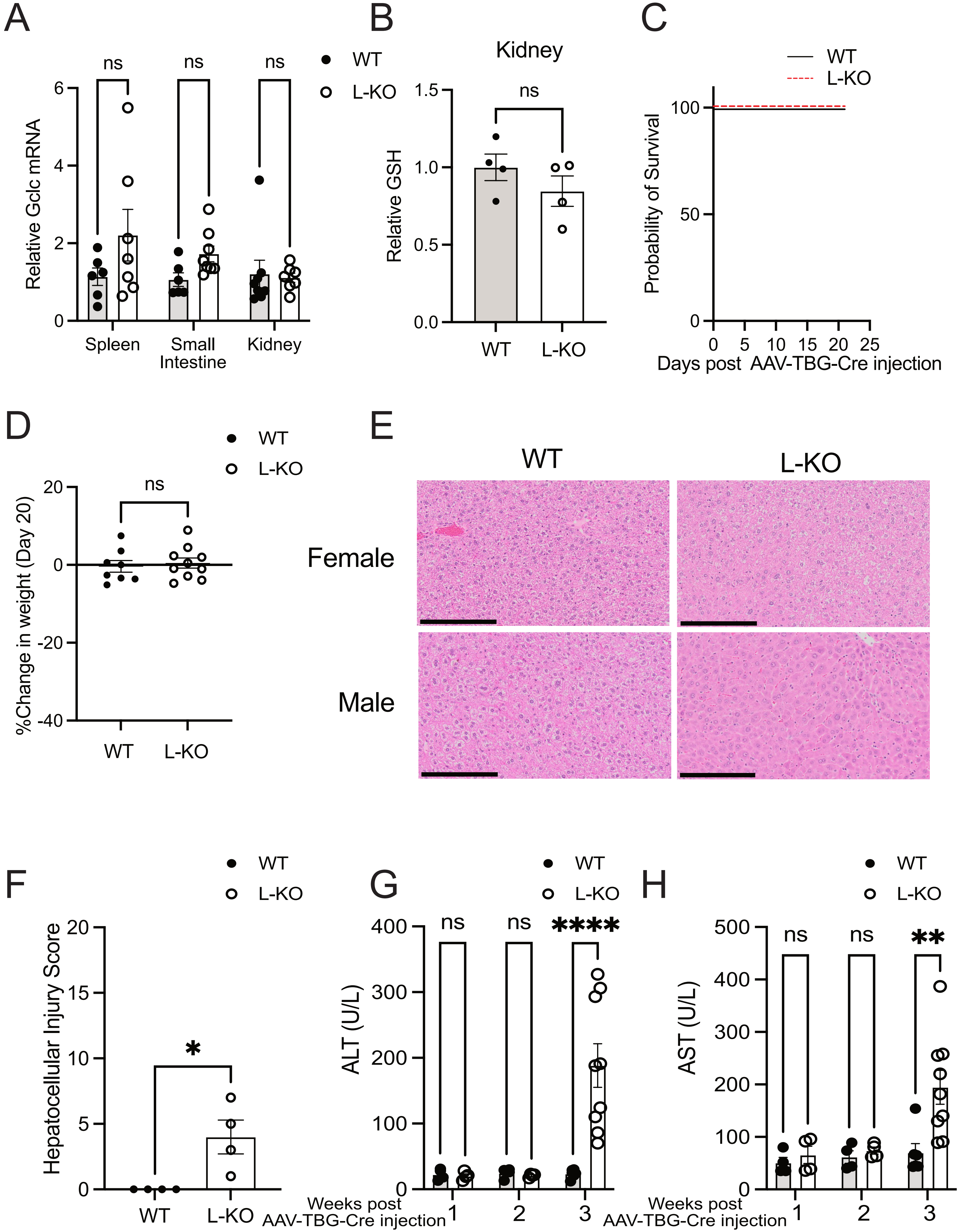

Figure S5. Inducible liver-specific *Gclc* deletion does not cause liver failure in mice. Related to Figure 4.

(A) Relative expression of *Gclc* mRNA in the spleen, small intestine, and kidney from (WT: *n* (Spleen & Small Intestine) =6, *n*(Kidney) = 8; and L-KO *n(*Spleen & Kidney) =7, n(Small Intestine) = 8) mice. Expression levels were normalized to the expression of the reference gene *Rps9*. A two-way ANOVA with subsequent Šidák's multiple comparisons test was used to determine statistical significance (WT vs. L-KO: Spleen P value = 0.1305, Small Intestine P value = 0.4773, Kidney P value = 0.9960)

(B) Relative abundance of GSH in the kidney of WT (*n*=4) and L-KO (*n*=4) mice following treatment with AAV-TBG-Cre. An unpaired two-tailed t-test was used to determine statistical significance (WT vs. L-KO P value = 0.2835)

(C) Percent survival of WT (*n*=6) and L-KO (*n*=6) mice following treatment with AAV-TBG-Cre.

(D) Percent change in body weight of WT (*n*=8) mice and L-KO (*n*=10) mice at day 20 following treatment with AAV-TBG-Cre. An unpaired two-tailed t-test was used to determine statistical significance (WT vs. L-KO P value = 0.6702)

(E) Representative H&E-stained slides of the liver of female WT and L-KO mice three weeks following treatment with AAV-TBG-Cre. Scale bars = 200 µm. Data shown is representative of at least 3 replicates.

(F) Hepatocellular Injury Score from H&E-stained slides of female WT(*n*=4) and L-KO (*n*=4) mice following treatment with AAV-TBG-Cre. An unpaired two-tailed t-test was used to determine statistical significance (WT vs. L-KO P value = 0.0212)

(G-H) Annotated serum biomarkers of liver damage in WT (*n*=6) and L-KO (*n*=9) mice in 1-3 weeks following treatment with AAV-TBG-Cre. A two-way ANOVA with subsequent Šidák's multiple comparisons test was used to determine statistical significance (WT vs. L-KO: (G): 1 week P value > 0.9999, 2 weeks P value = 0.9996, 3 weeks P value < 0.0001; (H): 1 week P value = 0.9787, 2 weeks P value = 0.9887, 3 weeks P value = 0.0017)

Indicated *n* values represent biologically independent samples from mice. Data are shown as mean ±SEM. ns = not significant, * P value < 0.05, ** P value < 0.01, *** P value < 0.001, **** P value < 0.0001.

Figure S6_related to Figure 4

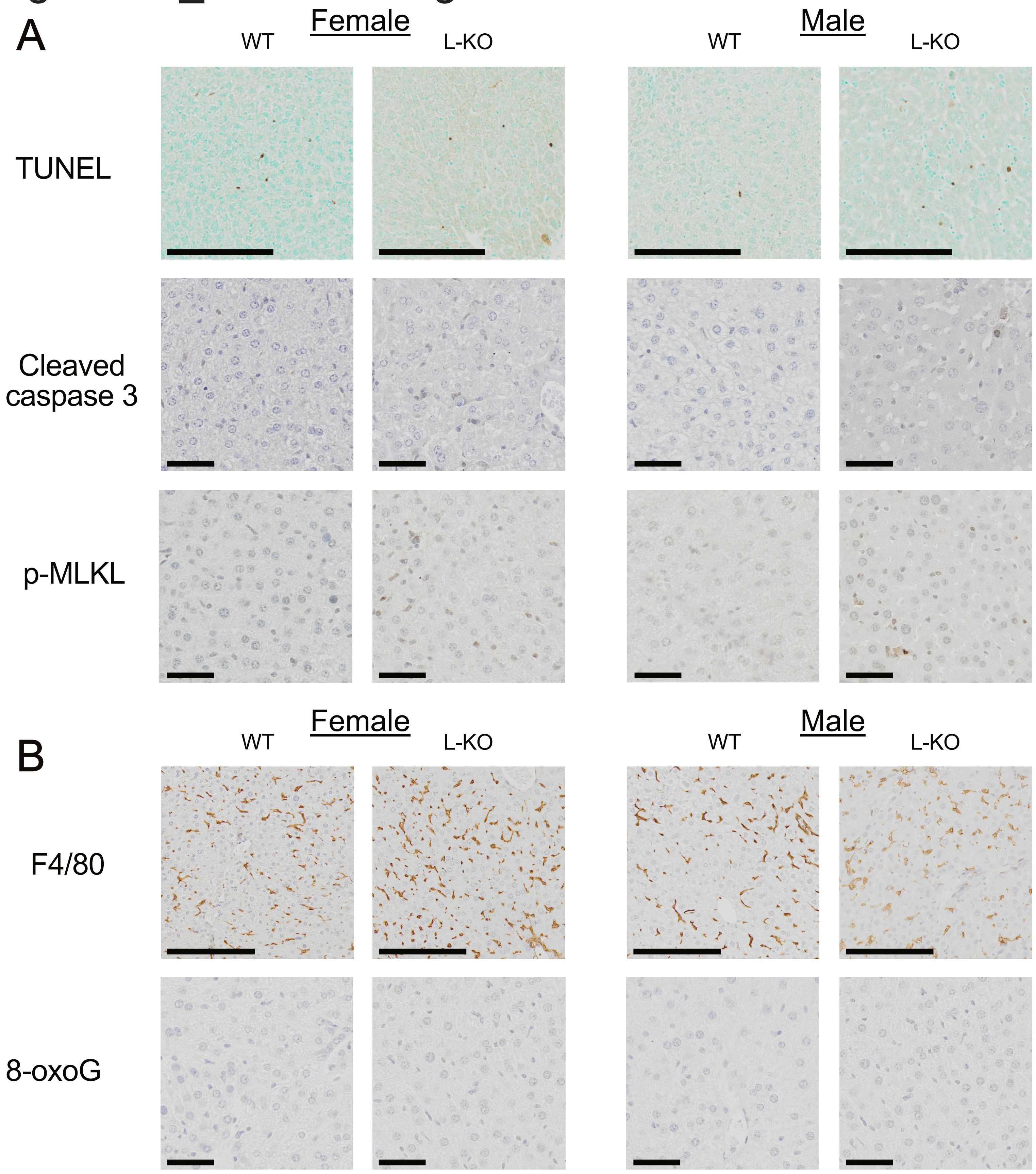

Figure S6. Liver Gclc deletion does not cause inflammation, oxidative stress, or apoptosis but increases necroptosis markers. Related to Figure 4.

(A) Representative immunohistochemical stains of markers of apoptosis (TUNEL and Cleaved Caspase 3) and necroptosis (p-MLKL) in the liver of WT and L-KO mice three weeks following treatment with AAV-TBG-Cre. Scale bars = 200 μm (TUNEL), 50 μm (Cleaved Caspase 3 and p-MLKL). Data shown is representative of at least 3 replicates.

(B) Representative immunohistochemical stains of F4/80 (a marker of macrophages) and 8-oxoguanine (8-oxoG; a marker of oxidative DNA damage) in the liver of WT and L-KO mice three weeks following treatment with AAV-TBG-Cre. Scale bars = 200 μm (F4/80), 50 μm (8-oxoguanine). Data shown is representative of at least 3 replicates.

Figure S7_related to Figure 4

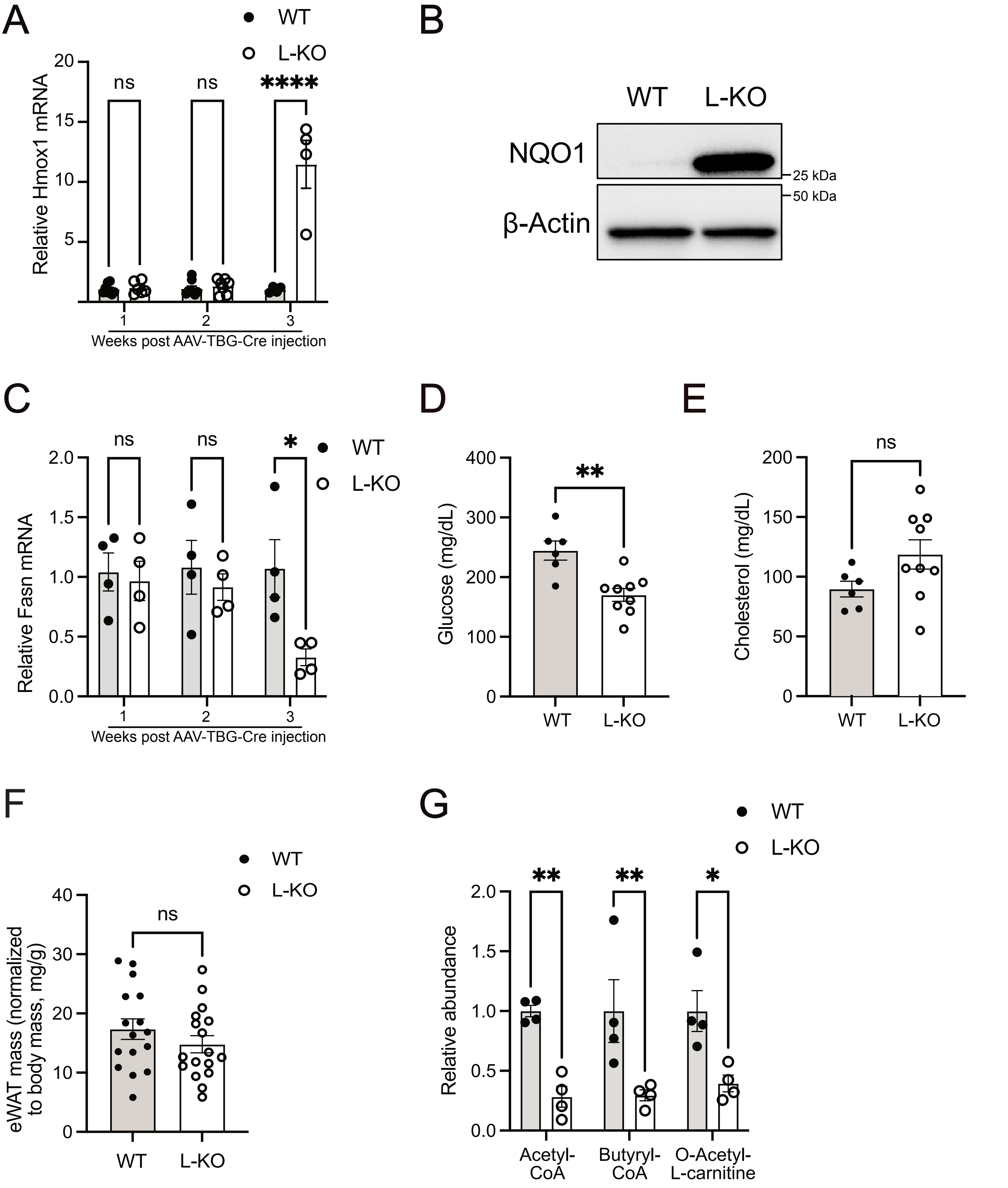

Figure S7. GSH produced in the liver promotes lipid synthesis and suppresses NRF2 activity. Related to Figure 4.

(A) Relative expression of *Hmox1* mRNA in the liver of WT (*n*=4-8) and L-KO (*n*=4-7) mice in 1-3 weeks following treatment with AAV-TBG-Cre. Expression levels were normalized to the expression of the reference gene *Rps9*. A two-way ANOVA with subsequent Šidák's multiple comparisons test was used to determine statistical significance (WT vs. L-KO: 1 week P value = 0.9936, 2 weeks P value = 0.9816, 3 weeks P value < 0.0001)

(B) Representative immunoblot analysis of NQO1 in the liver of WT and L-KO mice following treatment with AAV-TBG-Cre. Data shown is representative of at least 3 replicates.

(C) Relative expression of *Fasn* mRNA in the liver of WT (*n*=6) and L-KO (*n*=9) mice following treatment with AAV-TBG-Cre. Expression levels were normalized to the expression of the reference gene *Rps9*. A two-way ANOVA with subsequent Šidák's multiple comparisons test was used to determine statistical significance (WT vs. L-KO: 1 week P value = 0.9869, 2 weeks P value = 0.8787, 3 weeks P value = 0.00206).

(D-E) Serum concentration of (D) Glucose and (E) Cholesterol in WT (*n*=7) and Gclc L-KO (*n*=9) mice following treatment with AAV-TBG-Cre. An unpaired two-tailed t-test was used to determine statistical significance (WT vs. L-KO: (D) P value = 0.0016; (E) P value = 0.0956).

(F) Epididymal fat adipose tissue (eWAT) mass normalized to body mass from WT (*n*=16), Gclc L-KO (*n*=17) mice. An unpaired two-tailed t-test was used to determine statistical significance (WT vs. L-KO: P value = 0.2650)

(G) Relative abundance of annotated lipid precursors in the liver of WT (*n*=4) and L-KO (*n*=4) mice following treatment with AAV-TBG-Cre. A two-way ANOVA with subsequent Šidák's multiple comparisons test was used to determine statistical significance (WT vs. L-KO: Acetyl-CoA P value = 0.0053, Butyryl CoA P value = 0.0061, O-Acetyl- L-carnitine P value = 0.0186)

Indicated *n* values represent biologically independent samples from mice. Data are shown as mean ±SEM. An unpaired two-tailed t-test was used in (D-F). A two-way ANOVA with subsequent

Tukey's multiple comparisons test was used for (A), (C) and (G) to determine statistical significance. ns = not significant, * P value < 0.05, ** P value < 0.01, *** P value < 0.001, **** P value < 0.0001.

Figure S8_related to Figure 4

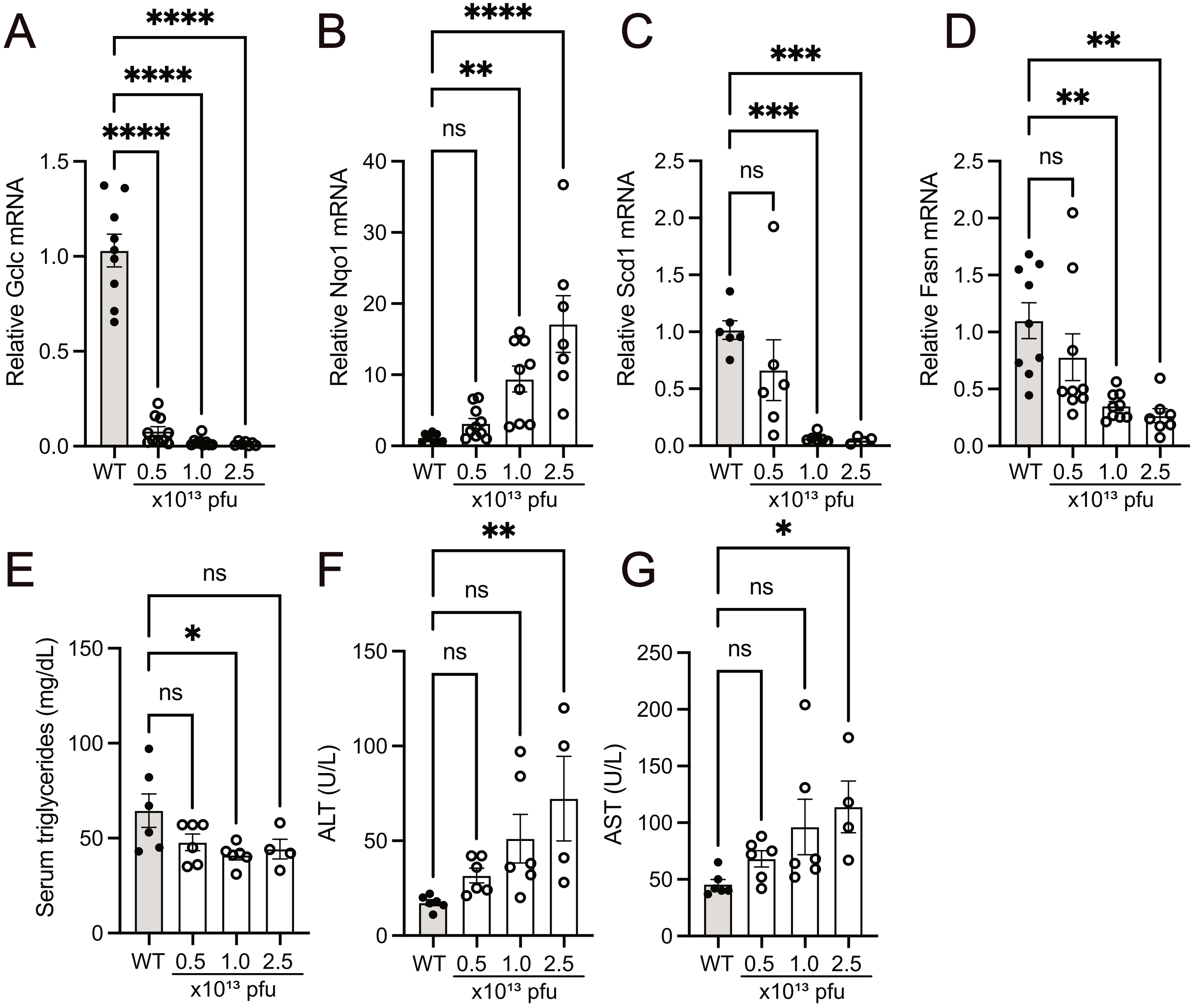

Figure S8. GSH produced in the liver promotes triglyceride abundance and suppresses NRF2 activity. Related to Figure 4.

(A-D) Relative expression of (A) *Gclc,* (B) *Nqo1,* (C) *Scd1* and (D) *Fasn* mRNA in the liver from WT (*n*=9) and L-KO mice treated with varying AAV-TBG-Cre titre (0.5 (*n*=10), 1.0 (*n*=9), 2.5 (*n*=7),) x10^{13} pfu. Expression levels were normalized to the expression of the reference gene *Rps9*. A one-way ANOVA with subsequent Dunnett's multiple comparisons test was used to determine statistical significance ((A): WT vs. 0.5x10^{13} pfu P value < 0.0001, WT vs. 1.0x10^{13} pfu P value < 0.0001 WT vs. 2.5x10^{13} pfu P value < 0.0001; (B): WT vs. 0.5x10^{13} pfu P value = 0.7723, WT vs. 1.0x10^{13} pfu P value = 0.0095 WT vs. 2.5x10^{13} pfu P value < 0.0001; (C): WT vs. 0.5x10^{13} pfu P value = 0.2536, WT vs. 1.0x10^{13} pfu P value = 0.0007, WT vs. 2.5x10^{13} pfu P value = 0.0017; (D): WT vs. 0.5x10^{13} pfu P value = 0.2534, WT vs. 1.0x10^{13} pfu P value = 0.0016, WT vs. 2.5x10^{13} pfu P value = 0.0010)

(E-G) Serum levels of (E) Triglycerides, (F) ALT and (G) AST in WT (*n*=6) and L-KO mice treated with varying AAV-TBG-Cre titre (0.5 (*n*=6), 1.0(*n*=6), 2.5(n=*4*)) x10^{13} pfu. A one-way ANOVA with subsequent Dunnett's multiple comparisons test was used to determine statistical significance ((E): WT vs. 0.5x10^{13} pfu P value = 0.1239, WT vs. 1.0x10^{13} pfu P value = 0.0223, WT vs. 2.5x10^{13} pfu P value = 0.0881; (F): WT vs. 0.5x10^{13} pfu P value = 0.6507, WT vs. 1.0x10^{13} pfu P value = 0.0811, WT vs. 2.5x10^{13} pfu P value = 0.0092; (G): WT vs. 0.5x10^{13} pfu P value = 0.6287, WT vs. 1.0x10^{13} pfu P value = 0.0863, WT vs. 2.5x10^{13} pfu P value = 0.0336).

Indicated *n* values represent biologically independent samples from mice. Data are shown as mean ±SEM. ns = not significant, * P value < 0.05, ** P value < 0.01, *** P value < 0.001, **** P value < 0.0001.

Figure S9_related to Figure 4

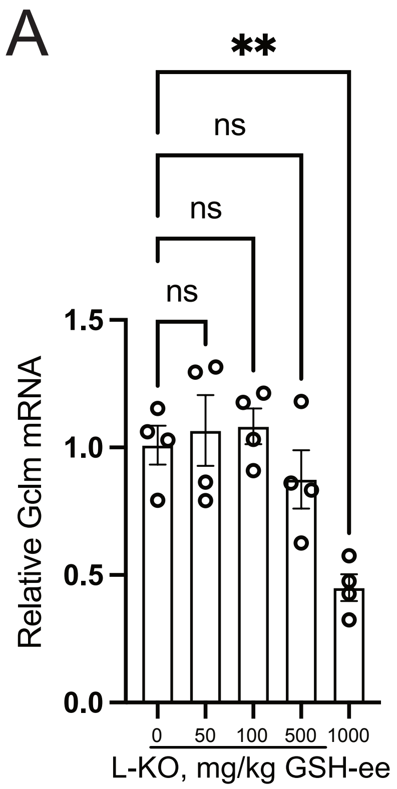

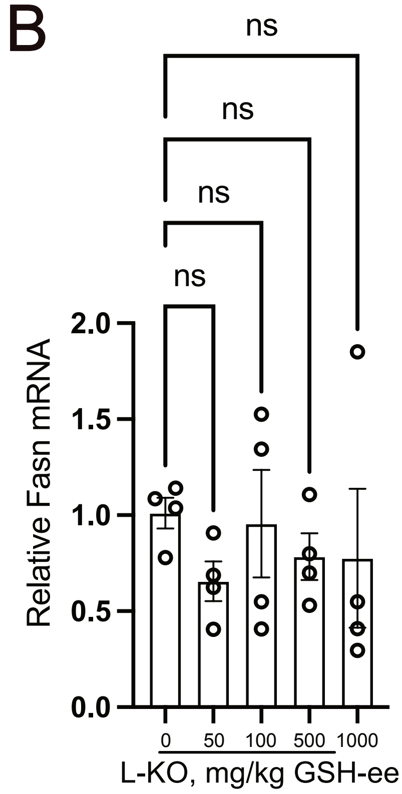

Figure S9. GSH maintains lowered NRF2 activity in the liver. Related to Figure 4.

(A-B) Relative expression of (A) *Gclm,* and (B) *Fasn* mRNA in the liver from L-KO (*n*=4 per group) mice treated with varying doses of GSH-ee (0, 50, 100, 500, and 1000 mg/kg) following AAV-TBG-Cre injection Expression levels were normalized to the expression of the reference gene *Rps9*. A one-way ANOVA with subsequent Dunnett's multiple comparisons test was used to determine statistical significance ((A): 0 mg/kg GSH-ee vs. 50 mg/kg GSH-ee P value = 0.9788, 0 mg/kg GSH-ee vs. 100 mg/kg GSH-ee P value = 0.9511, 0 mg/kg GSH-ee vs. 500 mg/kg GSH-ee P value = 0.7215, 0 mg/kg GSH-ee vs. 1000 mg/kg GSH-ee P value = 0.0032; (B): 0 mg/kg GSH-ee vs. 50 mg/kg GSH-ee P value = 0.6232, 0 mg/kg GSH-ee vs. 100 mg/kg GSH-ee P value = 0.9993, 0 mg/kg GSH-ee vs. 500 mg/kg GSH-ee P value = 0.8757, 0 mg/kg GSH-ee vs. 1000 mg/kg GSH-ee P value = 0.8625)

Indicated *n* values represent biologically independent samples from mice. Data are shown as mean ±SEM. ns = not significant, * P value < 0.05, ** P value < 0.01, *** P value < 0.001, **** P value < 0.0001.

Figure S10_related to Figure 5

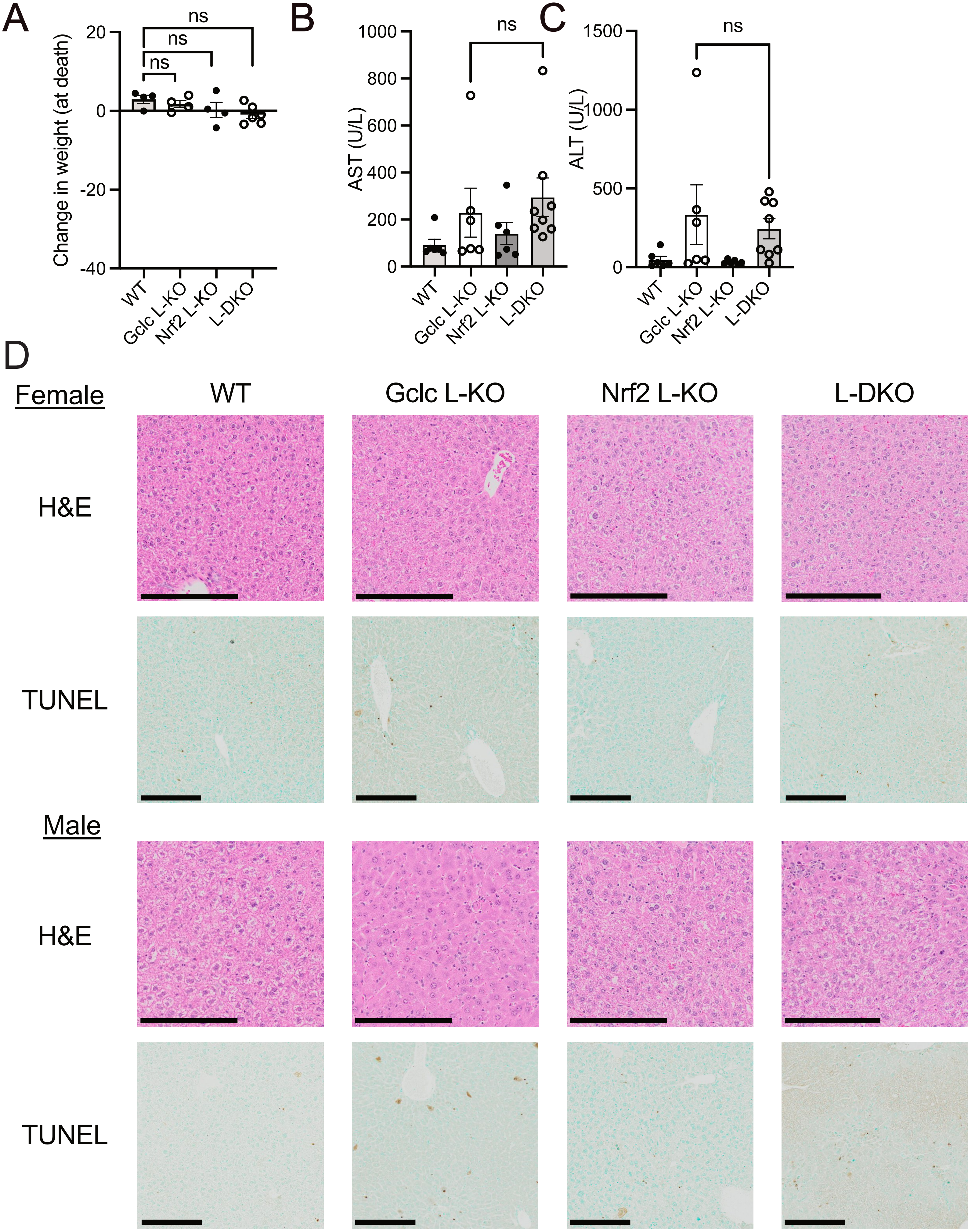

Figure S10. Combined *Gclc* and *Nrf2* deletion in the liver does not cause liver failure at acute time points. Related to Figure 5.

(A) Percent change in weight of WT (*n*=4), Gclc L-KO (*n*=4), Nrf2 L-KO (*n*=4), and L-DKO (*n*=6) mice three weeks following treatment with AAV-TBG-Cre. A one-way ANOVA with subsequent Dunnett's multiple comparisons test was used to determine statistical significance (WT vs. Gclc L-KO P value = 0.8459, WT vs. Nrf2 L-KO P value = 0.3429, WT vs. L-DKO P value = 0.0917)

(B-C) Serum biomarkers of liver damage; (B) AST and (C) ALT in WT (*n*=6), Gclc L-KO (*n*=6), Nrf2 L-KO (*n*=6) and L-DKO (*n*=8) mice three weeks following treatment with AAV-TBG-Cre. A one-way ANOVA with subsequent Šidák's multiple comparisons test was used to determine statistical significance (B): Gclc L-KO vs. L-DKO: P value = 0.5239; (C): Gclc L-KO vs. L-DKO: P value = 0.5006)

(D) Representative H&E-stained slides and immunohistochemical stains of TUNEL (marker of apoptosis) of the liver from female and male WT, Gclc L-KO, Nrf2 L-KO, and L-DKO mice three weeks following treatment with AAV-TBG-Cre. Scale bars = 200 μm. Data shown is representative of at least 3 replicates.

Indicated *n* values represent biologically independent samples from mice. Data are shown as mean ±SEM. ns = not significant, * P value < 0.05, ** P value < 0.01, *** P value < 0.001, **** P value < 0.0001.

Figure S11_related to Figure 5

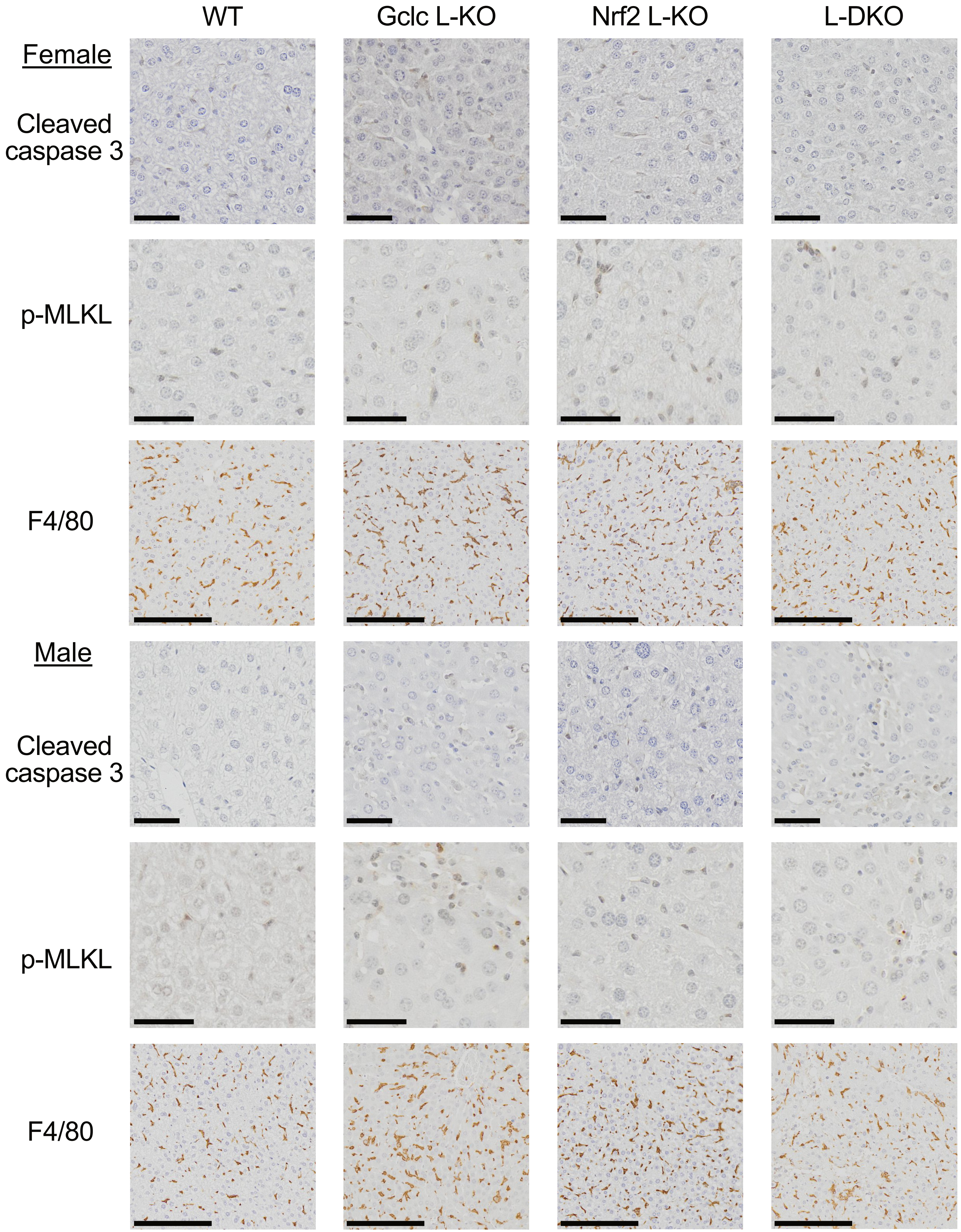

Figure S11. Combined *Gclc* and *Nrf2* deletion in the liver does not cause inflammation or cell death at acute time points. Related to Figure 5.

Representative immunohistochemical stains of Cleaved Caspase 3 (a marker of apoptosis), p-MLKL (a marker of necroptosis) and F4/80 (a marker of macrophages) in the liver of WT and L-KO female and male mice three weeks following treatment with AAV-TBG-Cre. Scale bars = 50 µm (Cleaved Caspase 3 and p-MLKL) and 200 µm (F4/80). Data shown is representative of at least 3 replicates.

Figure S12_related to Figure 5

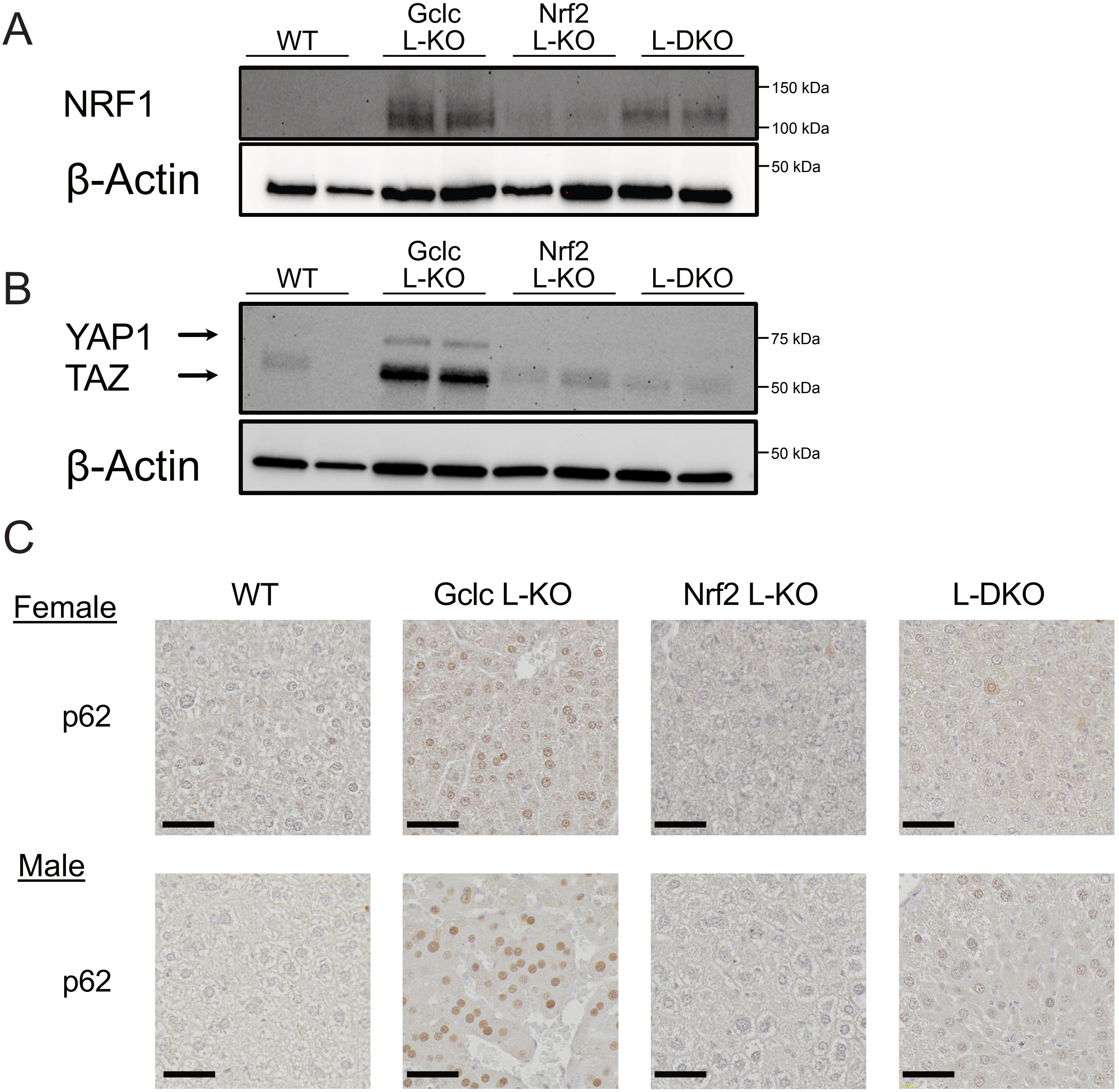

Figure S12. Induction of fibrotic and autophagic markers in liver-specific Gclc KO mice is dependent on Nrf2 activation. Related to Figure 5.

(A) Immunoblot analysis of NRF1 in the liver of WT, Gclc L-KO, Nrf2 L-KO, and L-DKO mice following treatment with AAV-TBG-Cre.

(B) Immunoblot analysis of YAP1/TAZ in the liver of WT, Gclc L-KO, Nrf2 L-KO, and L-DKO mice following treatment with AAV-TBG-Cre.

(C) Representative immunohistochemical stains of p62 (a marker of fibrosis) in the liver of male and female WT, Gclc L-KO, Nrf2 L-KO, and L-DKO mice three weeks following treatment with AAV-TBG-Cre. Scale bars = 50μm. Data shown is representative of at least 3 replicates.

Figure S13_related to Figure 5

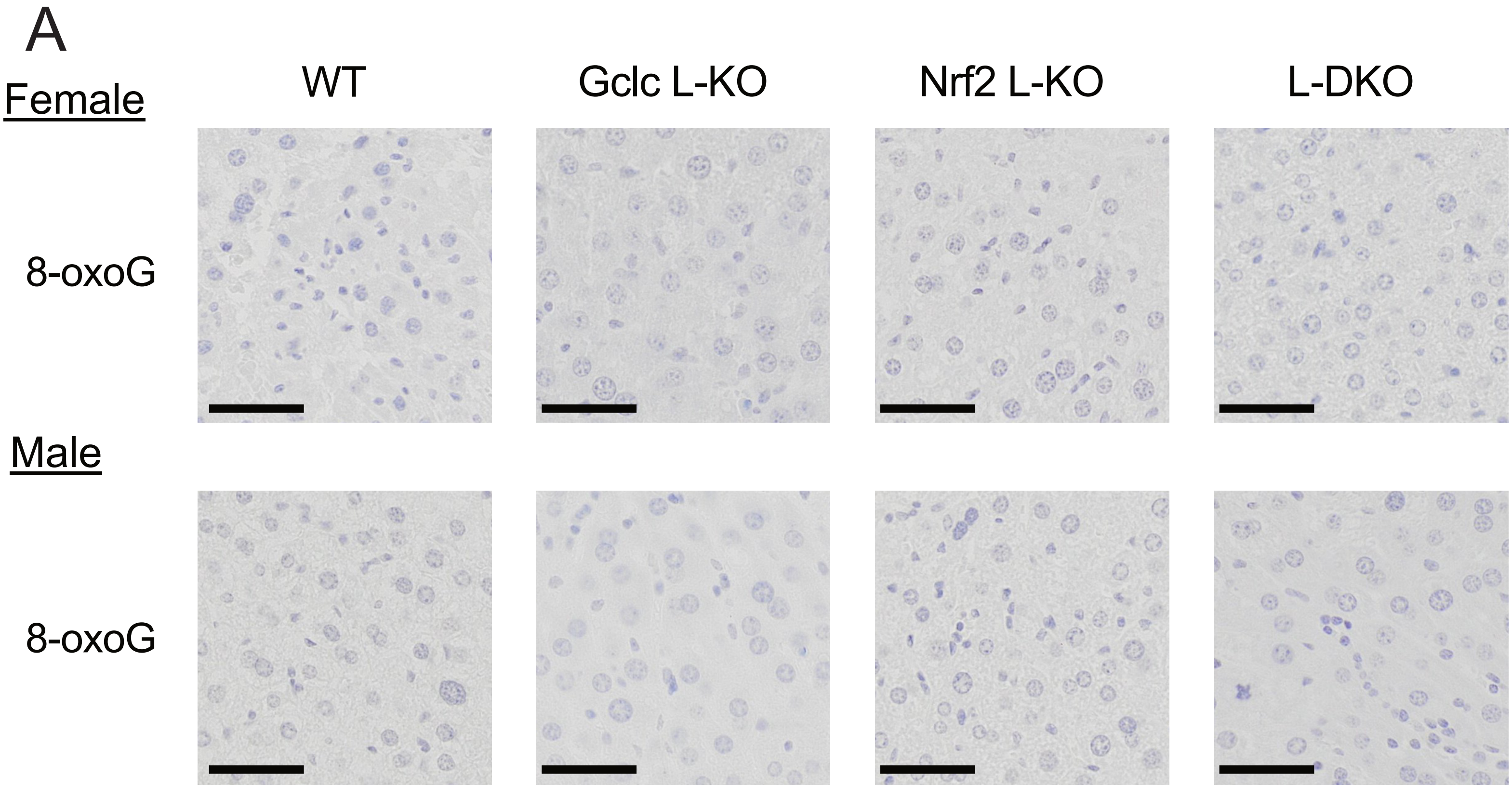

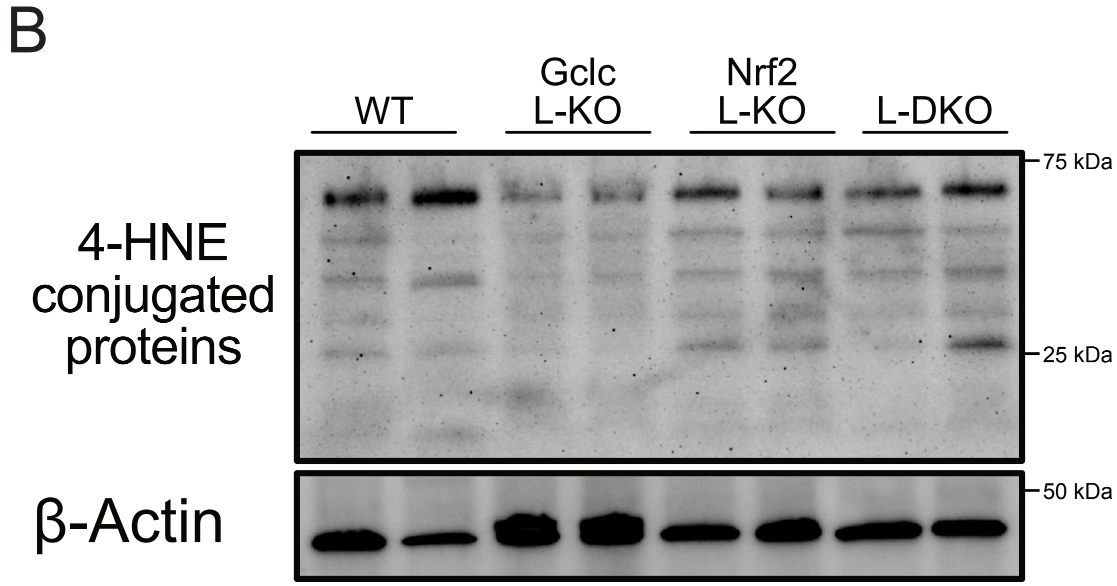

Figure S13. Combined *Gclc* and *Nrf2* deletion in the liver does not cause oxidative stress at acute time points. Related to Figure 5.

(A) Representative immunohistochemical stains of 8-oxoguanine (8-oxoG; a marker of oxidative DNA damage) in the liver of female and male WT and L-KO mice three weeks following treatment with AAV-TBG-Cre. Scale bars = 50 µm. Data shown is representative of at least 3 replicates.

(B) Immunoblot analysis of 4-HNE conjugated proteins in the liver of WT, Gclc L-KO, Nrf2 L-KO, and L-DKO mice following treatment with AAV-TBG-Cre.

Figure S14_related to Figure 5

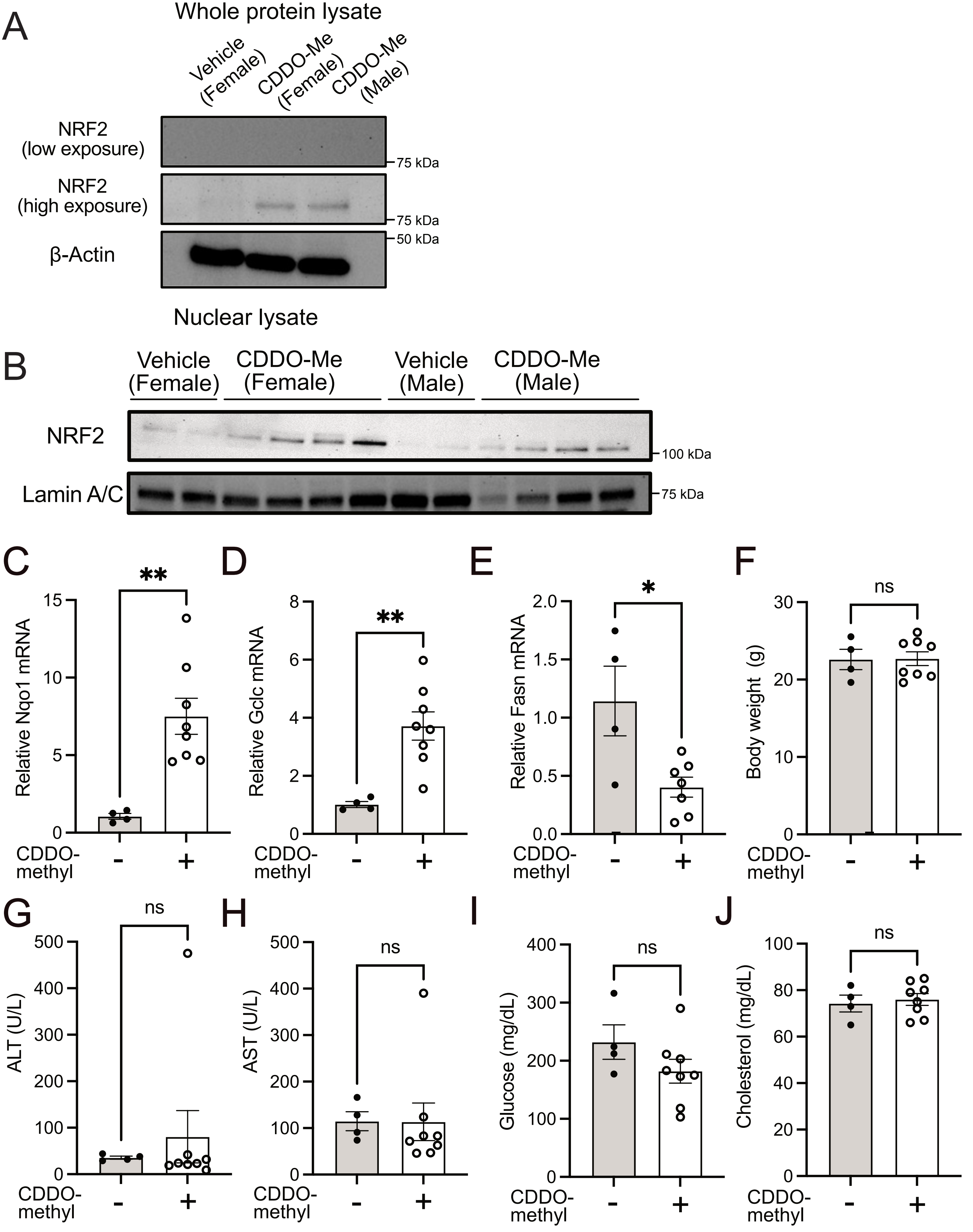

Figure S14. NRF2 activation via KEAP1 inhibition does not increase NRF2 activity to the degree observed in liver-specific *Gclc* deletion. Related to Figure 5.

(A-B) Immunoblot analysis of NRF2 in (A) whole-protein liver lysate (B) nuclear-protein liver lysate from WT, Gclc L-KO, Nrf2 L-KO, and L-DKO mice

(C-E) Relative mRNA expression of (C) *Nqo1*, (D) *Gclc* and (E) *Fasn* in the liver of WT mice without AAV-TBG-Cre injection following a 4-day treatment with either vehicle (*n*=4) or CDDO-methyl (*n*=7-8). Expression levels were normalized to the expression of the reference gene *Rps9*. An unpaired two-tailed t-test was used to determine statistical significance (Vehicle vs. CDDO-methyl: (C) P value = 0.0034; (D) P value = 0.0034; (E) P value = 0.0146).

(F) Body weight of WT mice without AAV-TBG-Cre injection following a 4-day treatment with either vehicle (*n*=4) or CDDO-methyl (*n*=8). An unpaired two-tailed t-test was used to determine statistical significance (Vehicle vs. CDDO-methyl P value = 0.9464).

(G-J) Concentration of annotated serum chemistry panel (G) ALT, (H) AST, (I) Glucose and (J) Cholesterol in WT mice treated with either vehicle (*n*=4) or CDDO-methyl (*n*=8). An unpaired two-tailed t-test was used to determine statistical significance (Vehicle vs. CDDO-methyl: (G) P value = 0.5954; (H) P value = 0.9838; (I) P value = 0.1894; (J) P value = 0.7010).

Indicated *n* values represent biologically independent samples from mice. Data are shown as mean ±SEM. An unpaired two-tailed t-test was used in (C-J) to determine statistical significance. ns = not significant, * P value < 0.05, ** P value < 0.01, *** P value < 0.001, **** P value < 0.0001.

Figure S15_related to Figure 6

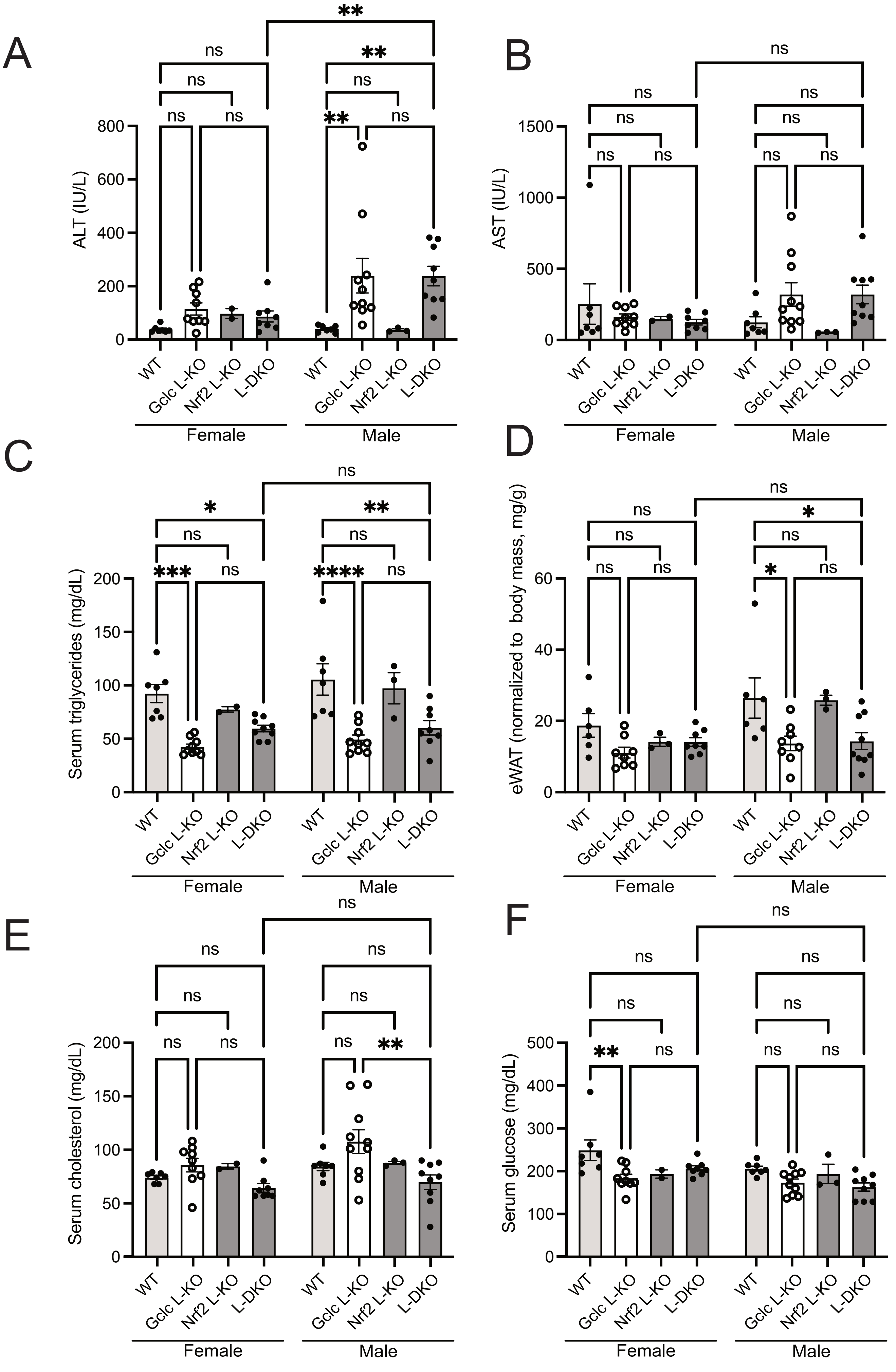

Figure S15. Combined liver-specific deletion of *Gclc* and *Nrf2* induces sex-dependent metabolic alterations at extended time points. Related to Figure 6.

(A-C) Serum biomarkers of liver damage; (A) ALT, (B) AST and (C) serum triglycerides in female and male WT, Gclc L-KO, Nrf2 L-KO, and L-DKO mice 10 weeks following treatment with AAV-TBG-Cre. A two-way ANOVA with subsequent Tukey's multiple comparisons test was used to determine statistical significance ((A): Female: WT vs. Gclc L-KO P value = 0.4995, WT vs. Nrf2 L-KO P value = 0.9027, WT vs. L-DKO P value = 0.8201, Gclc L-KO vs. L-DKO P value = 0.9500; Male: WT vs. Gclc L-KO P value = 0.0024, WT vs. Nrf2 L-KO P value > 0.9999, WT vs. L-DKO P value = 0.0033, Gclc L-KO vs. L-DKO P value > 0.9999; Female L-DKO vs. Male L-DKO P value = 0.0055; (B): Female: WT vs. Gclc L-KO P value = 0.7981, WT vs. Nrf2 L-KO P value = 0.9178, WT vs. L-DKO P value = 0.6160, Gclc L-KO vs. L-DKO P value = 0.9844; Male: WT vs. Gclc L-KO P value = 0.2101, WT vs. Nrf2 L-KO P value = 0.9565, WT vs. L-DKO P value = 0.2267, Gclc L-KO vs. L-DKO P value > 0.9999; Female L-DKO vs. Male L-DKO P value = 0.0516; (C): Female: WT vs. Gclc L-KO P value = 0.0003, WT vs. Nrf2 L-KO P value = 0.9811, WT vs. L-DKO P value = 0.0407, Gclc L-KO vs. L-DKO P value = 0.6114; Male: WT vs. Gclc L-KO P value < 0.0001, WT vs. Nrf2 L-KO P value = 0.9987, WT vs. L-DKO P value = 0.0017, Gclc L-KO vs. L-DKO P value = 0.9446; Female L-DKO vs. Male L-DKO P value >0.9999)

(D) Epididymal fat adipose tissue (eWAT) mass normalized to body mass from female and male WT, Gclc L-KO, Nrf2 L-KO, and L-DKO mice. Measurements were taken ten weeks post-treatment with AAV-TBG-Cre. A two-way ANOVA with subsequent Tukey's multiple comparisons test was used to determine statistical significance ((A): Female: WT vs. Gclc L-KO P value = 0.4948, WT vs. Nrf2 L-KO P value = 0.9830, WT vs. L-DKO P value = 0.9209, Gclc L-KO vs. L-DKO P value = 0.9897; Male: WT vs. Gclc L-KO P value = 0.0344, WT vs. Nrf2 L-KO P value > 0.9999, WT vs. L-DKO P value = 0.0426, Gclc L-KO vs. L-DKO P value > 0.9999; Female L-DKO vs. Male L-DKO P value > 0.9999)

(E-F) Serum (E) Cholesterol and (F) Glucose levels in female and male WT, Gclc L-KO, Nrf2 L-KO, and L-DKO mice 10 weeks following treatment with AAV-TBG-Cre. A two-way ANOVA with subsequent Tukey's multiple comparisons test was used to determine statistical significance ((E): Female: WT vs. Gclc L-KO P value = 0.9417, WT vs. Nrf2 L-KO P value = 0.9980, WT vs. L-DKO P value = 0.9817, Gclc L-KO vs. L-DKO P value = 0.3813; Male: WT vs. Gclc L-KO P value = 0.2885, WT vs. Nrf2 L-KO P value > 0.9999, WT vs. L-DKO P value = 0.8353, Gclc L-KO vs. L-DKO P value = 0.0038; Female L-DKO vs. Male L-DKO P value = 0.9992; (F): Female: WT vs. Gclc L-KO P value = 0.0062, WT vs. Nrf2 L-KO P value = 0.4254, WT vs. L-DKO P value = 0.2247, Gclc L-KO vs. L-DKO P value = 0.8396; Male: WT vs. Gclc L-KO P value = 0.4750, WT vs. Nrf2 L-KO P value = 0.9993, WT vs. L-DKO P value = 0.1795, Gclc L-KO vs. L-DKO P value = 0.9971; Female L-DKO vs. Male L-DKO P value = 0.1381).

Indicated *n* values represent biologically independent samples from mice. For all graphs, WT (*n*(Female) =7, *n*(Male) =7); Gclc L-KO (*n*(Female) =9, *n*(Male) =10); Nrf2 L-KO *n*(Female) =2, *n*(Male) =3) and L-DKO (Female) =8, *n*(Male) =9). Data are shown as mean ±SEM. ns = not significant, * P value < 0.05, ** P value < 0.01, *** P value < 0.001, **** P value < 0.0001.

Figure S16_related to Figure 6

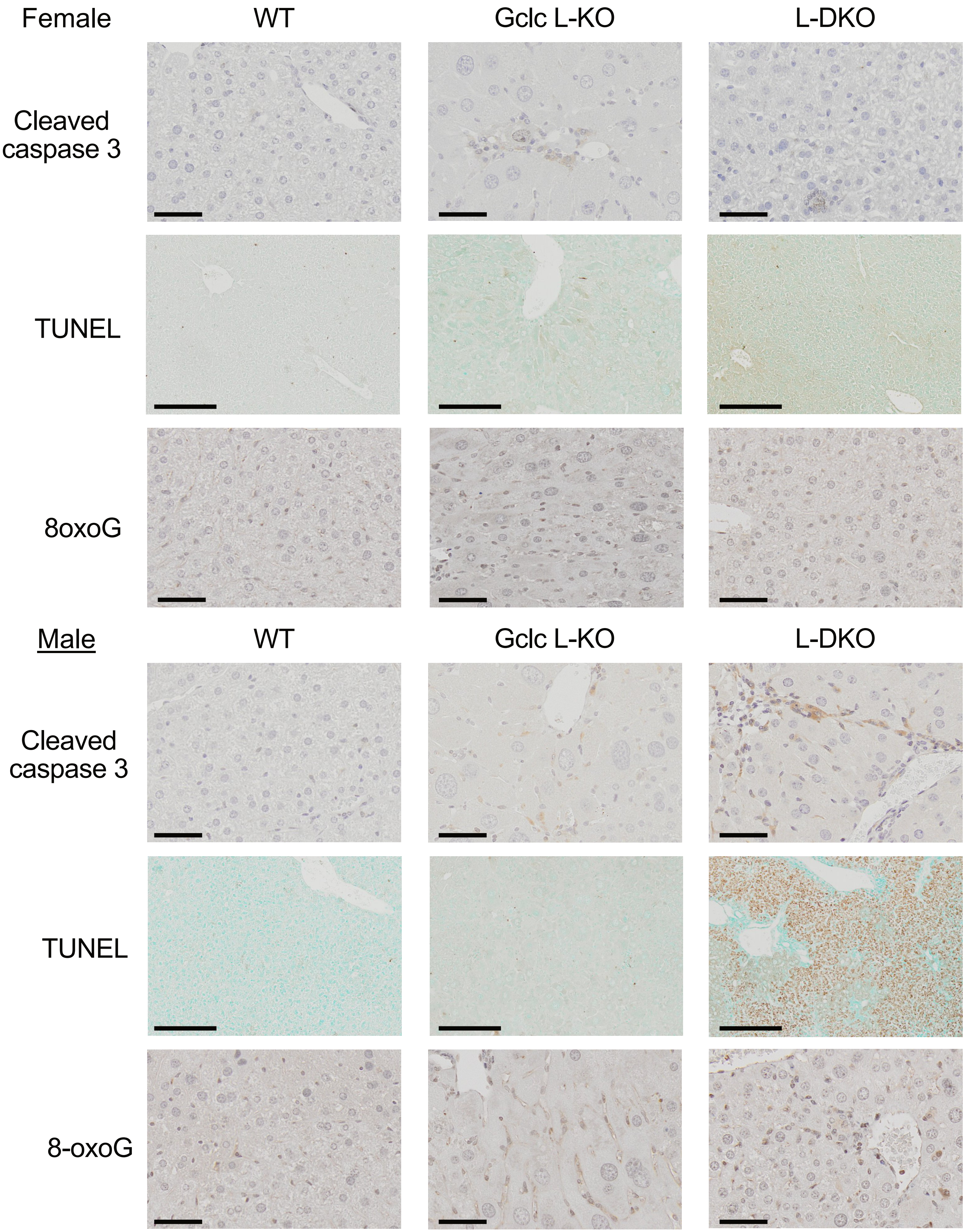

Figure S16. Combined liver-specific deletion of *Gclc* and *Nrf2* induces sex-dependent liver damage at extended time points. Related to Figure 6.

Representative immunohistochemical stains of cleaved-caspase 3 (top), TUNEL (middle) and 8-oxoguanine (8-oxoG; bottom) staining in the liver from female and male WT, Gclc L-KO, and L-DKO mice. Scale bars = 50µm (Cleaved caspase 3 and 8-oxoguanine) and 200µm (TUNEL). Data shown is representative of at least 3 replicates.

Figure S17_related to Figure 6

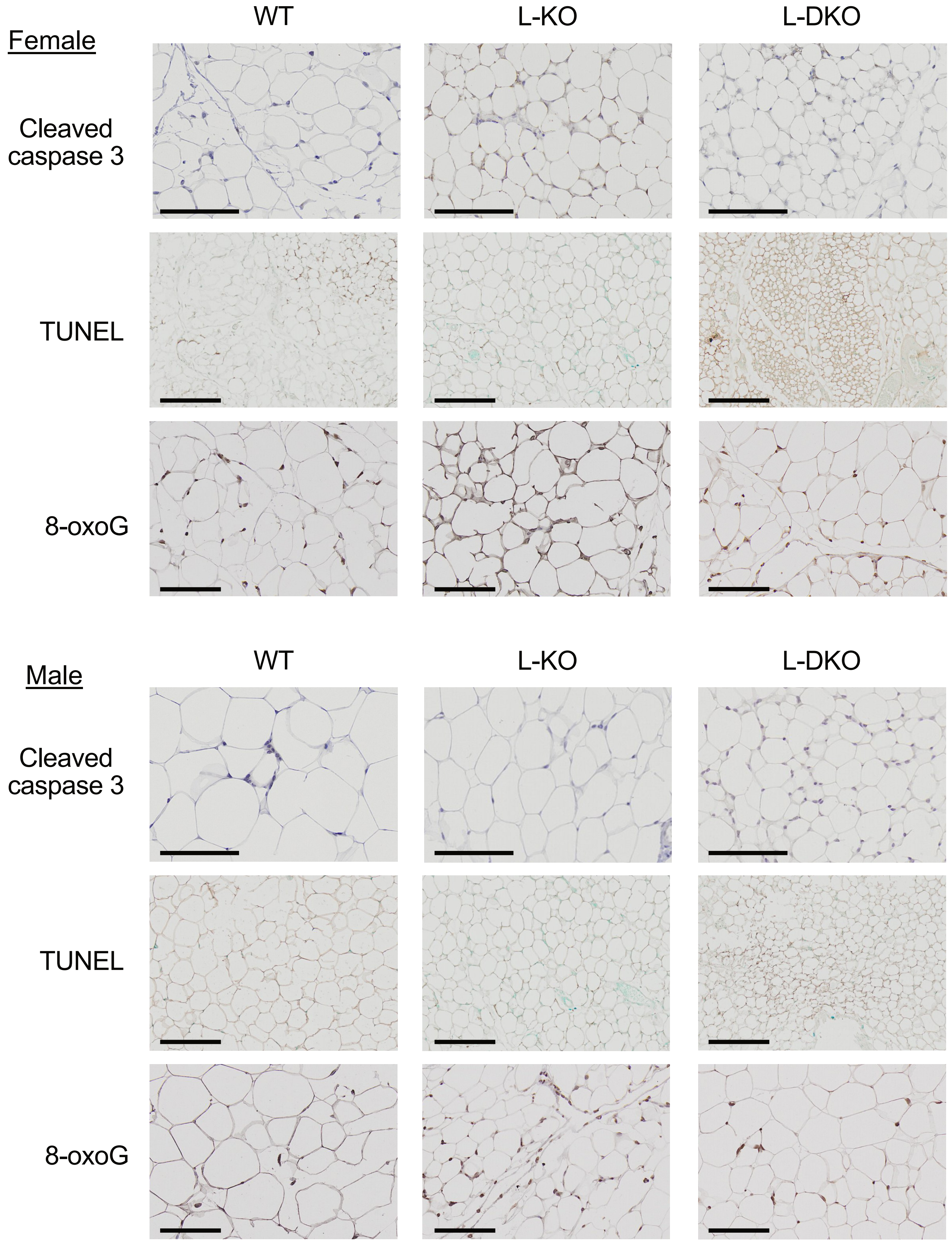

Figure S17. Combined liver-specific deletion of *Gclc* and *Nrf2* induces sex-dependent alterations in adipose tissue at extended time points. Related to Figure 6.

Representative immunohistochemical stains of cleaved-caspase 3 (top), TUNEL (middle) and 8-oxoguanine (8-oxoG; bottom) staining in the epididymal fat adipose tissue from female and male WT, L-KO and L-DKO mice. Scale bars = 50µm (8-oxoguanine), 100µm (Cleaved Caspase 3) and 200µm (TUNEL). Data shown is representative of at least 3 replicates.
