# Supplementary material for: Glutathione synthesis in the mouse liver supports lipid abundance through NRF2 repression
Source: Nat Commun. 2024 Jul 21;15:6152. doi: 10.1038/s41467-024-50454-2 (PMC11271484; doi:10.1038/s41467-024-50454-2)
Supplement: Supplementary file 3 — Description of Additional Supplementary Files [file 41467_2024_50454_MOESM3_ESM.pdf]

Description of Additional Supplementary Files

Supplementary Data 1. Related to Figure 2. Differential gene expression and gene set enrichment analysis of liver tissue from Gclc WT (*n*=4) and KO (*n*=4) mice. Indicated *n* values represent biologically independent samples from mice.

Supplementary Data 2. Related to Figure 2. Differential protein abundance for liver tissue from Gclc WT (*n*=4) and KO (*n*=4) mice. Indicated *n* values represent biologically independent samples from mice.

Supplementary Data 3. Related to Figure 3. Differential lipid abundance for adipose, liver, kidney, lung, and serum from Gclc WT (*n*=4) and KO (*n*=4) mice. Indicated *n* values represent biologically independent samples from mice.

Supplementary Data 4. Related to Figure S4. Differential gene expression and gene set enrichment analysis of liver tissue from Gclc WT (*n*=4) and liver-specific KO (*n*=4) mice. Indicated *n* values represent biologically independent samples from mice.

Supplementary Data 5. Related to Figure 5. Differential abundance (log2 fold change) for RNA, protein, and lipids for liver and serum from WT (*n*=4) compared to Gclc L-KO (*n*=4), Nrf2 L-KO (*n*=4), and L-DKO (*n*=6) mice. Indicated *n* values represent biologically independent samples from mice.

Supplementary Data 6. Related to METHODS. Primers used for quantitative PCR analysis.
