# Supplementary material for: Glutathione synthesis in the mouse liver supports lipid abundance through NRF2 repression
Source: Nat Commun. 2024 Jul 21;15:6152. doi: 10.1038/s41467-024-50454-2 (PMC11271484; doi:10.1038/s41467-024-50454-2)
Supplement: Supplementary file 10 — Reporting Summary [file 41467_2024_50454_MOESM10_ESM.pdf]

nature portfolio

Corresponding author(s): Isaac S Harris

Last updated by author(s): June 3, 2024

Reporting Summary

Nature Portfolio wishes to improve the reproducibility of the work that we publish. This form provides structure for consistency and transparency in reporting. For further information on Nature Portfolio policies, see our Editorial Policies and the Editorial Policy Checklist.

Statistics

For all statistical analyses, confirm that the following items are present in the figure legend, table legend, main text, or Methods section.

n/a	Confirmed	
☐	☒	The exact sample size (*n*) for each experimental group/condition, given as a discrete number and unit of measurement
☐	☒	A statement on whether measurements were taken from distinct samples or whether the same sample was measured repeatedly
☐	☒	The statistical test(s) used AND whether they are one- or two-sided<br>*Only common tests should be described solely by name; describe more complex techniques in the Methods section.*
☒	☐	A description of all covariates tested
☐	☒	A description of any assumptions or corrections, such as tests of normality and adjustment for multiple comparisons
☐	☒	A full description of the statistical parameters including central tendency (e.g. means) or other basic estimates (e.g. regression coefficient) AND variation (e.g. standard deviation) or associated estimates of uncertainty (e.g. confidence intervals)
☐	☒	For null hypothesis testing, the test statistic (e.g. *F*, *t*, *r*) with confidence intervals, effect sizes, degrees of freedom and *P* value noted<br>*Give P values as exact values whenever suitable.*
☐	☒	For Bayesian analysis, information on the choice of priors and Markov chain Monte Carlo settings
☒	☐	For hierarchical and complex designs, identification of the appropriate level for tests and full reporting of outcomes
☒	☐	Estimates of effect sizes (e.g. Cohen's *d*, Pearson's *r*), indicating how they were calculated

Our web collection on statistics for biologists contains articles on many of the points above.

Software and code

Policy information about availability of computer code

Data collection	IHC representative images were collected using OLYMPUS VS-ASW 2.9.2 and OlyVIA Ver.2.9, Global LC-MS metabolite peaks were manually identified and integrated by EL-Maven Version 0.11.0 and peaks from sulfur metabolite containing LCMS were integrated using Thermo Xcaliber Qual Browser. MS-DIAL was used to identify, and align lipid peaks from lipidomics experiments. ProLuCID algorithm (publicly available at http://fields.scripps.edu/yates/wp/?page_id=821), Mouse UniProt database (release 2017) and DTASelect (version 2.0) were used to identify the peptides from proteomics experiments. BioRender.com was used to generate all schematics.
Data analysis	Quantitative PCR data was analyzed with Thermo Fisher Design & Analysis Software v2.6.1. GraphPad Prism 9 and R version 3.3.2 and later were used to carry out statistical analysis on the data. Adiposoft plugin for Image J was used to quantify adipose cell sizes. Thermo Xcaliber Qual Browser was used to analyze peaks generated from metabolomic analyses. Gene set enrichment analysis was conducted with FGSEA an R package (http://bioconductor.org/packages/fgsea/). Peptide quantification was performed with reporter ion mass tolerance set to 30 ppm with Integrated Proteomics Pipeline (IP2).

For manuscripts utilizing custom algorithms or software that are central to the research but not yet described in published literature, software must be made available to editors and reviewers. We strongly encourage code deposition in a community repository (e.g. GitHub). See the Nature Portfolio guidelines for submitting code & software for further information.

Data

Policy information about availability of data

All manuscripts must include a data availability statement. This statement should provide the following information, where applicable:

- Accession codes, unique identifiers, or web links for publicly available datasets
- A description of any restrictions on data availability
- For clinical datasets or third party data, please ensure that the statement adheres to our policy

Data supporting these findings are included within the article and its supplementary material. Source data are provided with this paper. The mass spectrometry proteomics data have been deposited to the ProteomeXchange Consortium via the PRIDE103 partner repository with the dataset identifier PXD052674. The RNA-seq data generated in this study have been deposited in the Gene Expression Omnibus (GEO) database under accession codes GSE263190 and GSE263119.

Human research participants

Policy information about studies involving human research participants and Sex and Gender in Research.

Reporting on sex and gender	n/a
Population characteristics	n/a
Recruitment	n/a
Ethics oversight	n/a

Note that full information on the approval of the study protocol must also be provided in the manuscript.

Field-specific reporting

Please select the one below that is the best fit for your research. If you are not sure, read the appropriate sections before making your selection.

☒ Life sciences ☐ Behavioural & social sciences ☐ Ecological, evolutionary & environmental sciences

For a reference copy of the document with all sections, see nature.com/documents/nr-reporting-summary-flat.pdf

Life sciences study design

All studies must disclose on these points even when the disclosure is negative.

Sample size	Sample size of at least N=4 animals was used based on differences recorded in our pilot experiments.
Data exclusions	For lipidomic experiments, lipid species not fully identified by the MS2 spectra were excluded from analysis. No data was excluded from the rest of the experiments.
Replication	All experiments were replicated in at least 4 animals and done at least twice, except for transcriptomic/proteomic/metabolomic/lipidomic experiments.
Randomization	Mice of desired genotypes were both age matched and sex matched and assigned randomly to their treatment groups.
Blinding	Investigators were not blinded to group allocation during experiments due to technical limitations. All treatments and measurements were performed under the same conditions for all genotypes. Further, all samples were analyzed together and subjected to the same data processing.

Reporting for specific materials, systems and methods

We require information from authors about some types of materials, experimental systems and methods used in many studies. Here, indicate whether each material, system or method listed is relevant to your study. If you are not sure if a list item applies to your research, read the appropriate section before selecting a response.

Materials & experimental systems

n/a	Involved in the study
[ ]	[x] Antibodies
[x]	[ ] Eukaryotic cell lines
[x]	[ ] Palaeontology and archaeology
[ ]	[x] Animals and other organisms
[x]	[ ] Clinical data
[x]	[ ] Dual use research of concern

Methods

n/a	Involved in the study
[x]	[ ] ChIP-seq
[x]	[ ] Flow cytometry
[x]	[ ] MRI-based neuroimaging

Antibodies

Antibodies used

The antibodies used for the immunoblot assays were:
1. GCLC (Santa Cruz Biotech, #sc-390811)
2. NQO1 (Sigma Prestige Antibodies, HPA007308)
3. ACTIN (Sigma, A1978).
4. NRF2 (Cell Signaling Technology, #12721)
5. NRF1 (Cell Signaling Technology, #8052)
6. YAP/TAZ (Cell Signaling, #8418)
7. 4-HNE (Abcam, #ab46545)
8. Lamin A/C (Cell Signaling, #4777)
9. NQO1 (Abcam, #ab196196)
10. NRF2 (Abcam, # ab31163)
11. TUNEL (Abcam, #ab206386)
12. Cleaved Caspase 3(Cell Signaling, #9664)
13. F4/80 (Cell Signaling, #70076)
14. 8-oxoguanine (Abcam, #ab62623)
15. p62 (Cell Signaling, #23214)

Validation

The antibodies used are commercially available and have been validated by their respective manufacturers.

Animals and other research organisms

Policy information about studies involving animals; ARRIVE guidelines recommended for reporting animal research, and Sex and Gender in Research

Laboratory animals

12-18 weeks old Male and Female C57BL/6 mice with either WT, Gclc f/f, Gclc f/f R26CreERT2, Nrf2 f/f or Gclc f/f Nrf2 f/f genotypes.

Wild animals

No wild animals were used in this study.

Reporting on sex

Findings reported here apply to both sexes. Sex of mice were determined at 14-28 days using protocols approved by the University Committee on Animal Resources at the University of Rochester Medical Center. The mice assigned to each group were sex balanced and from our pilot studies, we found our phenotypes to be sex independent. Thus, we do not report on sex differences between mice.

Field-collected samples

Study did not involve samples collected from the field.

Ethics oversight

All animal studies were performed according to protocols approved by the University Committee on Animal Resources at the University of Rochester Medical Center.

Note that full information on the approval of the study protocol must also be provided in the manuscript.
